# The pivotal role of cytokines in liver disease pathogenesis and therapeutic potential

**DOI:** 10.3389/fimmu.2025.1694582

**Published:** 2025-11-17

**Authors:** Li Li, Xuhua Li, Fan Zhang

**Affiliations:** 1 Department of Hepatobiliary Pancreatic Oncology, Hefei Cancer Hospital, Chinese Academy of Sciences, Hefei, China; 2 Institute of Health and Medical Technology, Hefei Institutes of Physical Science, Chinese Academy of Sciences, Hefei, China

**Keywords:** cytokine, liver cirrhosis, hepatocellular carcinoma, inflammation, immunotherapy

## Abstract

Liver disease is a major threat to human health and life safety, primarily encompassing hepatitis caused by various factors (viral, alcoholic, fatty and autoimmune hepatitis), cirrhosis and liver cancer. Cytokines are molecules found on cell membranes that mediate the inflammation, immunity and a range of cellular functions, such as cell differentiation, proliferation, metastasis and apoptosis. In general, the delicate balance between pro- and anti-inflammatory effects is maintained by the body’s regulatory mechanisms. Once this balance is disturbed, complex chain reactions can occur, including systemic injury, tumor, multi-organ failure or death, resulting in the release of cytokines. This review mainly focuses on the classification, biological characteristics, pathogenesis, signaling pathways of various cytokines (interleukins, interferons, tumor necrosis factor, colony-stimulating factors, chemokines and growth factors) and their important roles in the occurrence and development of different liver diseases, mediating the immune responses, and further discusses the application prospects of cytokines in the clinical treatments of liver diseases.

## 1 Introduction

Pathogens, autoimmune diseases, genetic disorders, and malignant tumors have been identified as triggers for life-threatening systemic inflammatory syndromes, characterized by excessive activation of immune cells and release of cytokines (1, 2). This heightened immune response involves increased activity of dendritic cells (DC), lymphocytes, macrophages, and other immune cells, resulting in elevated levels of interleukins (IL) such as IL-6, IL-8, and IL-10, as well as C-reactive protein (CRP). These inflammatory markers contribute to the severity of systemic response (3, 4). The dysregulated inflammatory response initiates a self-reinforcing feedback loop that endangers the host’s life, a phenomenon recognized as cytokine release syndrome (CRS) or cytokine storm (CS) (5). CRS usually manifests with the symptoms of fever, fatigue, anorexia, hypotension, hypoxia, and even organ dysfunction (6). Early detection of cytokines is crucial for determining treatment strategies and predicting disease outcomes.

The inflammasome, an intracellular signaling complex of innate immune system, stimulated by the danger signals emitted from damaged cells and pathogens, resulting in the maturation and release of pro-inflammatory cytokines such as IL-1β, IL-18 and IL-37, causing the activation of cysteine proteases including caspase-1/4/5/8/11 (7, 8), ultimately triggering cell pyroptosis (9). Macrophages contribute to local inflammation by generating reactive oxygen species (ROS), secreting cytokines and chemokines (CKs), and attracting more immune cells (10, 11). The involvement of inflammasome leads to various aseptic inflammatory diseases, hereditary autoinflammatory diseases, metabolic disorders, cardiovascular diseases, neurodegenerative diseases and cancers (12). Previous studies have suggested that inflammatory mediator and transcription factors including ILs, CKs, tumor necrosis factor-α (TNF-α), transforming growth factor-β (TGF-β), and granulocyte macrophage colony-stimulating factor (GM-CSF), as well as nuclear factor kB (NF-kB), signal transducer and activator of transcription 3 (STAT3) are essential for cancer-related inflammation (13).

The primary liver cancer (PLC) ranks as the fourth common malignant tumors worldwide with a high mortality rate and it’s the leading cause of cancer-related death in China (14, 15). According to the pathological type of PLC, they’re divided into hepatocellular carcinoma (HCC), intrahepatic cholangiocarcinoma (ICC) and mixed liver cancer (16). Early-stage PLC usually doesn’t cause noticeable symptoms, while advanced PLC manifests as abdominal pain, distension, nausea, and poor appetite. The common treatments encompass surgery, ablation, transcatheter hepatic arterial chemoembolization (TACE), targeted therapy, immunotherapy and chemotherapy, but recurrence rate of post-surgery can be as high as 40%-70% within five years. HCC comprises approximately 75%-85% of PLC with poor prognosis. The critical pathogenesis of HCC including hepatitis virus B (HBV) or hepatitis virus C (HCV) infection, alcohol abuse and obesity (17, 18). Lipid alterations are a common consequence of chronic HBV and HCV infection, alcoholic hepatitis, nonalcoholic fatty liver disease (NAFLD), and steatohepatitis (19). Chronic hepatitis damages the liver epithelial cells, leading to DNA injury and genomic mutations, facilitating tumor cells evasion of immune surveillance and triggering the liver self-defense mediated by immune cells including natural killer (NK) cells, NKT cells and intrahepatic macrophages (20–23), which eventually causing liver fibrosis and HCC. There’re various pro-fibrogenic mediators including TGF-β1, platelet-derived growth factor (PDGF), endothelin-1 (ET-1), toll-like receptor 4 (TLR4) and reactive oxygen species (ROS), stimulating the epithelial to mesenchymal transition, resulting in the secretion of elastin, collagen, proteoglycans and glycoproteins, which play essential roles in liver fibrosis (24). ET-1, a peptide distributed in liver, inducing hepatic stellate cells proliferation, which is responsible for fibrosis/cirrhosis and portal hypertension (25). ROS is derived from molecular oxygen and formed by reduction–oxidation (redox) reactions (26). The glutathione, lipoic acid and taurine are pleiotropic molecules acting as ROS scavengers, which are involved in fibrosis progression by modulating the TGF-β, PDGF and TLR pathways (24).

Cytokines play a pivotal role in liver diseases by impacting inflammatory responses, hepatocyte proliferation, liver fibrosis or cirrhosis. Certain cytokines can contribute to inflammation and liver injury, they can also facilitate liver regeneration and enhance antiviral defenses (27–30). Comprehensive understanding of cytokines pleiotropy is essential for achieving optimal therapeutic outcomes in the management of liver disease. This review delves into the biological attributes of various cytokines and their diverse functions in the progression and treatments of liver diseases. Furthermore, innovative immunotherapy strategies utilizing cytokines as targets in liver diseases will be explored and discussed.

## 2 Classification and biological characteristics of cytokines

Cytokines are small proteins characterized by a broad spectrum of biological activities. They are synthesized and secreted by different immune cells (like monocytes, macrophages, T cells, B cells and NK cells) and non-immune cells (like endothelial cells, epidermal cells, and fibroblasts) in response to stimuli (31). Cytokines play critical roles in regulating innate and adaptive immunity, as well as in facilitating the tissue repair. By binding to specific receptors, they modulate cell growth and differentiation. Cytokines can be categorized into ILs, interferons (IFNs), TNF, colony-stimulating factors (CSF), CKs, and growth factors (GF) (32). Cytokines can act in an autocrine or paracrine manner, exhibiting both pro-inflammatory and anti-inflammatory effects.

**Figure 1:**
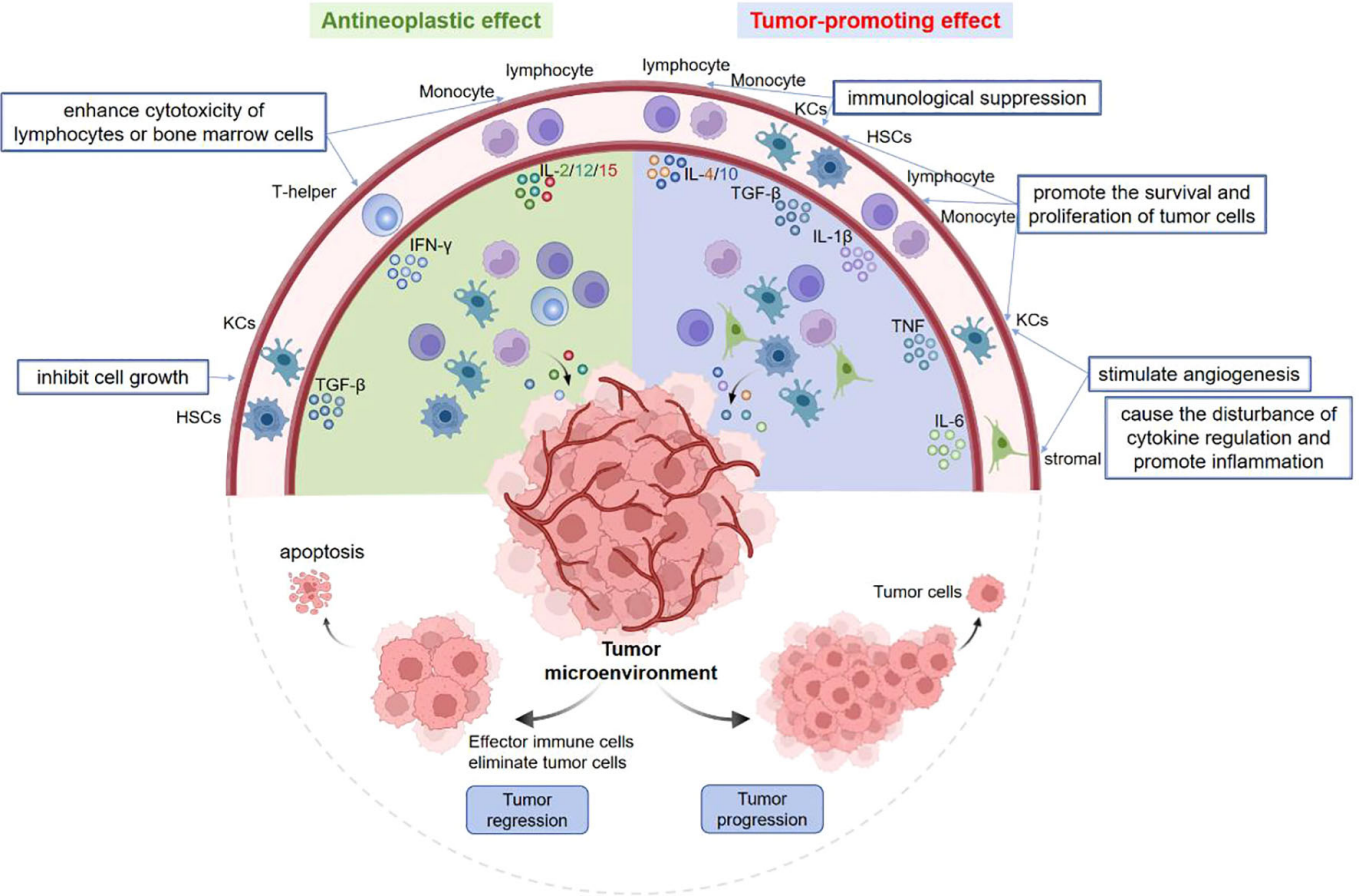
Pleiotropy of cytokines in tumor microenvironment. Some cytokines have antineoplastic and/or tumor-promoting effects. On the one hand, TGF-β can inhibit tumor cell growth, IFN-γ and IL-2/12/15 suppress cell proliferation by enhancing the cytotoxicity of lymphocytes or bone marrow cells. On the other hand, TGF-β, TNF, IL-1β promote tumor cell proliferation. TNF and IL-6 can stimulate the angiogenesis, disturb cytokine regulation and trigger inflammation. Moreover, IL-4/10 and TGF-β can induce the immunosuppression.

Within the tumor microenvironment (TME), cytokines serve as vital signaling proteins with diverse functions, which have antineoplastic and/or tumor-promoting effects on the occurrence and progression of tumors (**Figure 1**). On the one hand, TGF-β can directly inhibit the growth of tumor cells, while IFN-γ, IL-2, IL-12 and IL-15 enhance the cytotoxicity of lymphocytes or bone marrow cells to suppress the proliferation of tumor cells. On the other hand, TGF-β, TNF, IL-1β can promote the cell survival and proliferation. TNF and IL-6 may disrupt the cytokine regulation and trigger the inflammation in TME. In addition, IL-10, IL-4 and TGF-β have the ability to induce immunosuppression, whereas TNF, IL-6 and chemokines can stimulate the angiogenesis (33). In this review, we illustrate the characteristics, signaling pathways of cytokines in various liver diseases.

Some cytokines have antineoplastic and/or tumor-promoting effects. On the one hand, TGF-β can inhibit tumor cell growth, IFN-γ and IL-2/12/15 suppress cell proliferation by enhancing the cytotoxicity of lymphocytes or bone marrow cells. On the other hand, TGF-β, TNF, IL-1β promote tumor cell proliferation. TNF and IL-6 can stimulate the angiogenesis, disturb cytokine regulation and trigger inflammation. Moreover, IL-4/10 and TGF-β can induce the immunosuppression.

### 2.1 Interleukin

#### 2.1.1 Biological characteristics of IL

Based on the recognition sequence homology and receptor chain similarity of interleukins, they’re divided into different families and exert important effects in inflammation, autoimmune diseases and cancers (34, 35) (**Table 1**). IL-1 and IL-6 family members are introduced detailedly in this review. IL-1 family consists of three main types: agonists (IL-1α, IL-1β, IL-18, IL-33, IL-36α, IL-36β and IL-36γ), antagonists (IL-1Ra, IL-36Ra and IL-38) and anti-inflammatory cytokine (IL-37) (53). They serve as crucial signaling molecules in both innate and adaptive immune systems, mediating the inflammatory responses to varieties of stimuli (54). The high-resolution structures of IL-1α, IL-1β, IL-1Ra, IL-18, IL-33, IL-36γ, IL-37 and IL-38 have been determined by X-ray crystallography or solution nuclear magnetic resonance (NMR). All of them have conserved β-trilobate conformation and a hydrophobic core consisting of 12β-sheets (55). In the context of tumor development and therapy, IL-1 expression is differentially regulated in tumor cells, tissue stromal cells and immune cells in a stage-specific and tissue-specific manner. IL-1 family members and their receptors have pleiotropic functions depending on the target cells, playing complicated roles in inflammation, tumorigenesis, tumor metastasis, immunosuppression and immune surveillance (56). IL-1 signaling regulation lies on IL-1R1 and IL-1R2. IL-1R1 is involved in the differentiation, expansion and survival of Th17 cells, as well related to autoimmune disease (57). Most IL-1 family members form into signaling complexes by binding to their homologous receptors, such as IL-1RI (IL-1α and IL-1β), IL-33R (ST2) and IL-36R (IL-36α, IL-36β and IL-36γ). IL-1 receptor accessory protein (IL-1RAcP) commonly serves as shared secondary receptor, facilitating the formation of cytokine-receptor co- receptor complexes. Therefore, targeting to the IL-1RAcP can selectively inhibit the signaling transduction mediated by IL-1 family members, which provides a potential strategy for treating cancers (58).

**Table 1:** Classifications and clinical applications of IL family members.

IL family member	Main representative	Clinical disease	Author
IL-1 family	Pro-inflammatory cytokines (IL-1α, IL-1β, IL-18, IL-33, IL-36α, IL-36β, IL-36γ)	Breast cancer, colon cancer, head and neck cancer, lung cancer, pancreatic cancer and melanoma	Gelfo V, et al. (36) Akdis M, et al. (34)
	Anti-inflammatory cytokines (IL-1Ra, IL-36Ra, IL-37, IL-38)	Colorectal cancer, inflammatory bowel disease, atopic dermatitis	Sugiura K, et al. (37) Dang J, et al. (38) Mesjasz A, et al. (39)
IL-6 family	IL-6, IL-11, IL-31	Colorectal cancer, pancreatic cancer, non-small cell lung cancer	Johnson DE, et al. (40) Miura T, et al. (41) Naqash ARTA, et al. (42)
IL-10 family	IL-10, IL-19, IL-20, IL-22, IL-24, IL-26, IL-28, IL-29	Psoriasis, hepatitis, pancreatitis, graft versus host disease (GVHD), human T-cell lymphotropic virus type 1 (HTLV-1) infection	Ouyang W, et al. (43) Shefler I, et al. (44) Brites C, et al. (45)
IL-12 family	IL-12, IL-23, IL-27, IL-35	Inflammatory bowel disease, tuberculosis, malaria, influenza virus infection, pancreatic cancer, HCC	Verstockt B, et al. (46) Tait Wojno ED, et al. (47) Kourko O, et al. (48)
γ- chain cytokine family	IL-2, IL-4, IL-7, IL-9, IL-15, IL-21	Colorectal cancer, melanoma (MM), non-small cell lung cancer, rheumatoid arthritis, psoriasis, systemic lupus erythematosus	Ma S, et al. (49) Long D, et al. (50)
IL with chemokine activity	IL-8, IL-16	HCC, colon, pancreatic, breast and lung cancer, Crohn’s disease	Fousek K, et al. (51) Jørgensen AR, et al. (52)

Recent studies have highlighted the significance of IL-1 family members including IL-18, IL-33, IL-36, IL-37 and IL-38 in mediating the inflammation and immune responses. They’re tightly regulated by antagonists and anti-inflammatory cytokines under physiological and pathological conditions (53). Some evidence demonstrate that IL-1 family can influence the expression of vascular endothelial growth factor (VEGF) and fibroblast growth factor (FGF), two mediators which sustain tumor progression. Blocking the IL-1 signaling pathways may disrupt the recruitment of immature cells and inhibit tumor immune evasion (59). IL-1α and IL-1β are key downstream factors in intrinsic and extrinsic pathways linked to inflammation and malignant tumors. IL-1α can translocate to the nucleus, acting as a transcription factor that initiate the signaling transduction by binding to DNA and enhancing IL-8 expression. IL-1β, extensively studied in the autoinflammatory diseases, contributing to atherosclerosis and cancer progression (60). Experiments have suggested that antibodies blocking IL-1β can prevent cardiovascular events and reduce the incidence and mortality of lung cancer, emphasizing the importance of IL-1 and related family members (such as IL-33 and IL-18) in shaping the innate immunity and inflammation responses (61, 62). Recent researches have indicated that IL-1β signaling is relevant to cell death of hepatocytes (63–65).

**Figure 2:**
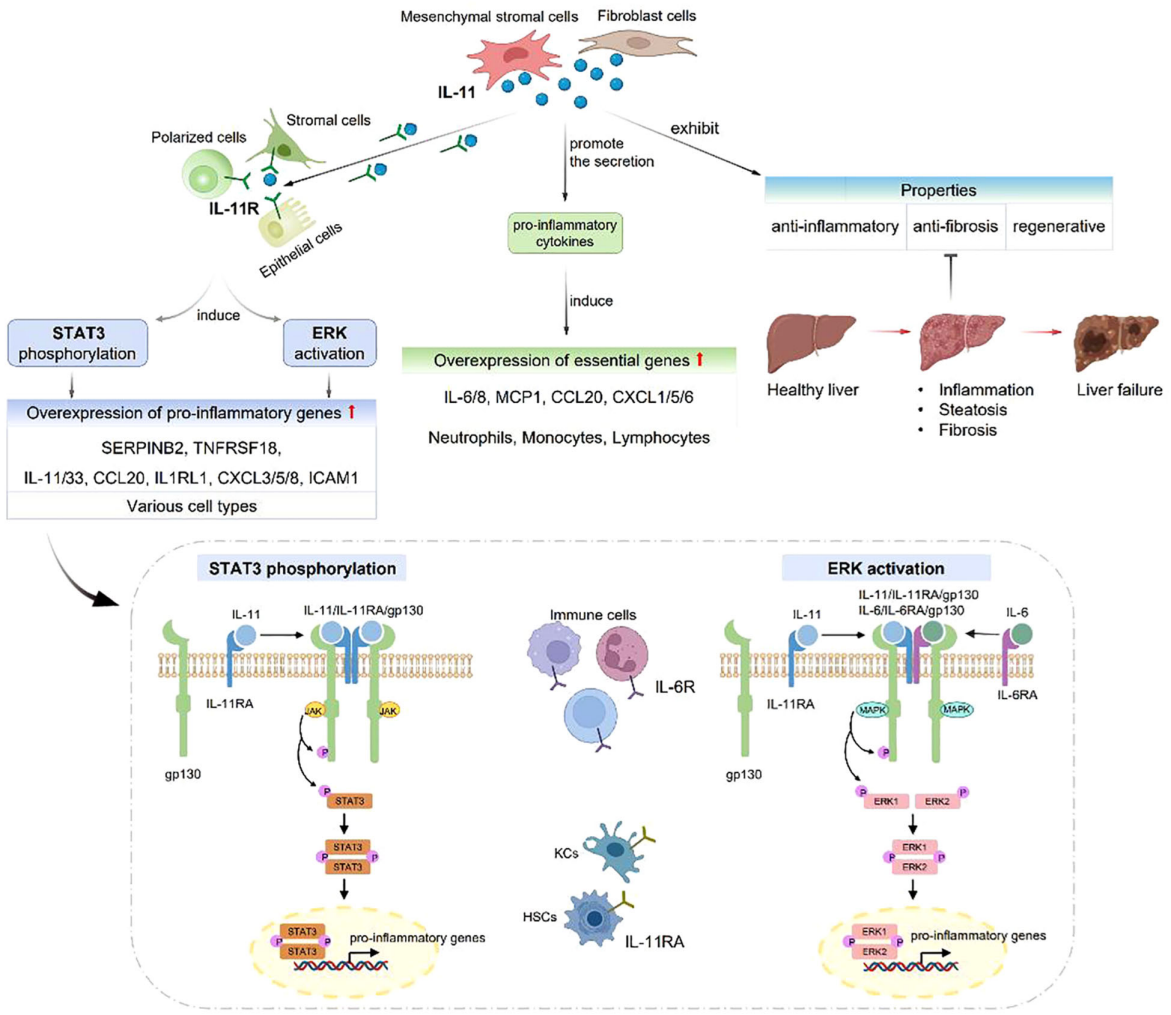
IL-11 plays distinct roles from IL-6 in biological and pathological aspects. IL-6R is expressed in immune cells, while IL-11R is expressed in stromal, epithelial and polarized cells. IL-11 can cause liver inflammation, steatosis, fibrosis and liver failure. IL-11 induces STAT3 phosphorylation and ERK activation, resulting in SERPINB2, TNFRSF18, IL-33, CCL20, IL1RL1, CXCL3/5/8, ICAM1 and IL-11 overexpression. IL-11 can also promote the secretion of IL-6, IL-8, MCP1, CCL20 and CXCL1/5/6.

IL-6 family proteins regulate various pathways through binding to gp130 receptor and affecting the liver regeneration. IL-11, a member of IL-6 family, playing distinct roles from IL-6 in biological and pathological aspects (**Figure 2**). IL-6R is predominantly expressed in the immune cells, while IL-11R is highly expressed in the stromal, epithelial and polarized cells, and IL-11RA is prominent in the hepatocytes and hepatic stellate cells. IL-11 can promote the development of liver diseases, eventually leading to inflammation, steatosis, fibrosis and liver failure. Widjaja et al. (66) reported that IL-11 induced the signaling cascade responses involving the transcriptional activator 3 (STAT3) phosphorylation and extracellular signal-regulated protein kinase (ERK) activation in various cell types, resulting in increased expression of pro-inflammatory genes, such as SERPINB2, TNFRSF18, IL-33, CCL20, IL1RL1, CXCL3/5/8, intercellular adhesion molecule 1 (ICAM1) and IL-11. Proteomic studies demonstrated that IL-11 promoted the secretion of pro-inflammatory cytokines, significantly increased the levels of IL-6, IL-8, monocyte chemoattractant protein 1 (MCP1), CCL20 and CXCL1/5/6, which were crucial for neutrophils, monocytes and lymphocytes. On the other hand, IL-11 was observed to exhibit anti-inflammatory, anti-fibrosis and regenerative properites (67).

IL-6R is expressed in immune cells, while IL-11R is expressed in stromal, epithelial and polarized cells. IL-11 can cause liver inflammation, steatosis, fibrosis and liver failure. IL-11 induces STAT3 phosphorylation and ERK activation, resulting in SERPINB2, TNFRSF18, IL-33, CCL20, IL1RL1, CXCL3/5/8, ICAM1 and IL-11 overexpression. IL-11 can also promote the secretion of IL-6, IL-8, MCP1, CCL20 and CXCL1/5/6.

#### 2.1.2 Signaling pathway of IL

IL-1 is an inflammatory cytokine that activates the genes associated with inflammation and immune diseases. The formation of IL-1 receptor heterodimer triggers a series of biological responses, including the activation of nuclear factor-κB (NF-κB) and mitogen-activated protein kinase (MAPK) pathways. IL-1 can bind to IL-1R on the cell surface, inducing downstream nuclear transcription such as NF-κB and activator protein-1 (AP-1) (68). This signaling cascade also involves a feedback regulatory mechanism that promotes the expression of inflammatory mediators like cyclooxygenase (COX) and nitric oxide synthase (NOS), ultimately leading to inflammation (69). IL-1 receptor-associated kinase 4 (IRAK4) is crucial in signaling pathways mediated by Toll-like receptor and IL-1R, which plays a key role in innate and adaptive immune responses (70, 71). MaIRAK4, a homologue of IRAK4, has been shown to modulate NF-κB pathway mediated by MaMyD88, leading to reduced expression of pro-inflammatory factors (IL-1β, IL-6, IL-8, TNF-α) when knocked down (72).

IL-6 activates the signaling molecule STAT3 by classical and trans-signaling pathways. Classical signaling occurs in the cells expressing IL-6Rα, inducing anti-inflammatory molecules, whereas transmembrane signaling happens in cells expressing gp130, triggering pro-inflammatory cytokine release and promoting chronic inflammation (73). Sun et al. (74) observed that mRNA let-7i regulated the maturation of dendritic cells (DC) targeting to IL-10 through janus kinase-signal transducers and activators of transcription (JAK-STAT3) pathway.

IL-17 is prominent for host immune defenses, tissue repair, inflammatory disease pathogenesis, and cancer progression (75). It can induce the expression of IL-1, IL-6, TNF, GM-CSF and CCL. The IL-23/IL-17 axis plays a crucial role in inflammatory responses (76). IL-23 induces the Th17 cells to secrete IL-17 by activating JAK-STAT cascade. Studies indicated that chronic inflammation could worsen cardiovascular complications in myeloproliferative neoplasms (MPNs) by activating JAK-STAT pathway (77). Liu et al. (78) verified that T cell immunoglobulin domain and mucin domain 4 (TIM-4) was a novel growth factor promoting non-small cell lung cancer (NSCLC) progression. IL-6 facilitated NSCLC metastasis by up-regulating TIM-4 expression through NF-κB pathway. Chen et al. (79) showed that IL-17A promoted the development of gallbladder cancer (GBC) through stimulating the epithelial-mesenchymal transformation (EMT) mediated by ERK/NF-κB pathway. IL-17A, served as a new therapeutic target and diagnostic marker, significantly impacting the treatment of GBC.

IL-33 is released by epithelial and smooth muscle cells of the airway system in response to environmental factors, such as allergens, viruses, parasites and pollutants (80). Recent studies have linked IL-33 to various cancers, including lung cancer, liver cancer, head and neck squamous cell carcinoma. The expression of IL-33/ST2 in cancer tissues correlates with tumor growth and progression (81, 82). IL-33 binds to ST2 on precursor mast cells, recruiting IL-1RAcP and activating a common signaling pathway mediated by Toll/interleukin-1 receptor (TIR). MyD88 recruitment further activates the ERK, JNK, p38 MAPK and NF-kB signaling pathways, resulting in the transcription of pro-inflammatory cytokines (such as IL-1β, IL-6, IL-13, TNF-α) and chemokines (83). The matrix metalloproteases (MMPs) belong to a family of zinc-dependent endopeptidases, regulated by cytokines and hormones, which are involved in cancer pathogenesis and progression (84). The tissue inhibitor of metalloproteinases (TIMPs) are multi-functional proteins mediating cellular signaling, among which TIMP-1, 2, 3, 4 are considered as natural inhibitors of metalloproteinases to facilitate cancer progression. TIMPs are endogenous protease inhibitors binding to MMPs to form complexes that regulate the activation or function of MMPs. The remodeling of myocardium extracellular matrix (ECM) modulated by TIMPs and MMPs is related to heart health and disease (85). IL-37 acts as an anti-inflammatory cytokine mediated by Rac1/NF-κB/MMP2 pathway (86). In 2021, Wang et al. (87) suggested that IL-37β was involved in endometrial cancer pathogenesis, offering a potential target for diagnosing and treating the endometrial cancer.

IL-1 superfamily members including IL-1α, IL-1β, IL-18, IL-36α, IL-36β, IL-36γ and IL-38, which play vital roles in inflammation, immunity and cancers. In synergy with IL-36Ra, IL-36 promotes the intestinal inflammation through the NF-KB pathway, which maintains the homeostasis by balancing the pro- and anti-inflammatory responses in the TME (88). IL-38 also exhibits anti-inflammatory property of decreasing IL-6, IL-1β, CCL5 and CXCL10 expression, reducing the production of pro-inflammatory mediators including IL-17 and IL-22 (89, 90), which is considered as a receptor antagonist in inhibiting IL-36 to bind to IL-36R (69). ILs play a crucial part in disease progression, which possess pro- or anti-inflammatory properties by mediating various signaling pathways (**Table 2**). However, further researches are needed to confirm these effects.

**Table 2:** Categories, receptors and mechanisms of ILs.

Cytokines	Signaling pathway	Effect	Receptor	Reference
IL-1 (IL-1α, IL-1β)	NF-κB pathway	proinflammatory effect	IL-1R	(72)
IL-6	NF-κB pathway	proinflammatory or anti-inflammatory effect	IL-6R	(73)
IL-10	JAK-STAT3 pathway	anti-inflammatory effect	IL-10R	(74)
IL-17	ERK/NF-κB pathway	proinflammatory effect	IL-17R	(76)
IL-18	NF-κB pathway	proinflammatory effect	IL-18R	(62)
IL-27	STAT1	anti-inflammatory effect	WSX-1/gp130	(48)
IL-33	ST2+Foxp3+/MyD88	proinflammatory effect	ST2	(81, 83)
IL-36	MAPKs and NF-κB pathway	proinflammatory or anti-inflammatory effect	IL-36R	(88)
IL-37	Rac1/NF-κB/MMP2 pathway	anti-inflammatory effect	IL-18R	(86, 87)
IL-38	MAPKs and NF-κB pathway	anti-inflammatory effect, similar to IL-1 receptor antagonist (IL-1Ra) and IL-36Ra	IL-1Rrp2, IL-36R	(69)

#### 2.1.3 Role of IL in liver diseases

NAFLD has emerged as a prevalent liver disorder worldwide (91). The expression levels of IL-1β, IL-18, procaspase-1 and nucleotide-binding oligomerization domain (NOD)-like receptor family - pyrin domain (NLRP3) are markedly increased in NAFLD. Hepatic stellate cells have the capability to engulf the NLRP3 particles, potentially enhancing IL-1β expression. This, in turn, leading to the pyroptosis and inflammasome release, finally causing the liver injury and fibrosis (63). As is known to all, IL-6 plays a pivotal role in immune responses at early stage of cancers. Current researches indicate that IL-6 impacts tumor cell renewal and metastasis by modulating downstream target named osteopontin (OPN), making it a prognostic risk factor of HCC (92).

Surgical resection is a primary treatment for HCC. However, the phenomenon of postoperative recurrence and metastasis is common in clinical practice, contributing to poor prognosis. The efficacy of current drug treatment for HCC is deemed unsatisfactory. Therefore, it’s urgent to explore novel therapies to improve the prognosis of patients and prevent the recurrence and metastasis of liver cancer after surgery. Moreover, rational approaches to prevention, surveillance, early detection, comprehensive diagnosis and treatments can increase the overall survival time of patients with HCC (93). Virus infection, especially hepatitis B virus (HBV) infection, is the leading cause of HCC (94, 95). So antiviral therapy is critical throughout the treatment process of HCC. Wang et al. (96) selected 92 HBeAg-positive patients with chronic hepatitis B (CHB) who received one year of standard long-acting interferon named IFN-α2b. Patients who did not respond to IFN therapy were treated with sequential low-dose IL-2 for 6 months. The regulatory T cells (Treg cells) number and programmed cell death protein 1 (PD-1) expression were reduced in non-responders. Sequential administration of IL-2 restored effective immune function, including signal transducers and transcriptional activator 1 (STAT1) activation. In addition, IL-2 therapy enhanced the frequency and function of HBV-specific CD8+ T cells, thereby improving the prognosis of patients. The constrain of IL-11-STAT3 signaling could effectively prevent the postoperative recurrence of HCC. Overall, this study suggested that sequential IL-2 therapy could be a promising approach to effectively save the immune function in patients with CHB who didn’t not respond to standard IFN therapy.

The liver expresses IL-1α, IL-33 and IL-18 in steady state, while under pathological conditions, IL-1 family members are up-regulated. Acute liver failure (ALF) is a life-threatening clinical syndrome with rapid hepatocyte injury and hepatic encephalopathy (97). Studies observed that IL-1α and IL-1β were related to the NF-kB signaling activation and liver damage, playing a central role inALF pathogenesis (98). Anakinra, a recombinant IL-1Ra, was verified to improve survival of patients with acute liver injury by regulating inflammatory response (99). It was confirmed that IL-33 served as an “alarmin” released by stressed hepatocytes, which could bind to the receptor termed suppressor of tumorigenicity 2 (ST2), then activated NF-kB and mitogenactivated protein kinases (MAPKs) pathways, ultimately promoting the formation of liver fibrosis (100, 101).

IL-36 exerts effects on cells and tissue by activating NF-kB, MAPKs, JNK, and ERK1/2 kinase cascades (102). Hu et al. (103) found that higher expression of IL-36 indicated better prognosis and longer survival of HCC. IL-37, an anti-inflammatory and antineoplastic cytokine, blocking the effects of IL-1α/β and IL-18, thereby inducing the autophagia and facilitating hepatocytes apoptosis in HCC through inhibiting the PI3K/Akt/mTOR signaling pathway (104). The interaction between pro-inflammatory and anti-inflammatory cytokines is a significant factor in host protection against the HCC progression. However, further researches are essential to investigate the complicated mechanisms involved and therapeutic potential of targeting these cytokines in HCC.

### 2.2 Interferon

#### 2.2.1 Classification of IFN

IFNs are important immune mediators in the natural defense against infectious agents and they’ re classified into types I, II and III based on the receptors they bind to (105). Type I IFNs are expressed in placental mammals, including IFN-α, IFN-β and IFN-ϵ. While IFN-δ and IFN-τ are found in non-primate and non-rodent mammals. IFNs exert anti-viral effects due to pro-inflammatory and anti-inflammatory properties (106, 107). Type III IFN (IFN-λ) has a limited action spectrum, with its primary function of providing an effective first defense line for mucosal surfaces (108, 109).

Type I and type III IFNs have unique ability to activate the STAT. leading to STAT1/STAT2 heterodimer formation, then interacting with IFN regulatory factor 9 (IRF9) to assemble into IFN-stimulated gene factor 3 (ISGF3) (110). NAFLD and non-alcoholic steatohepatitis (NASH) are significant challenges threatening human health due to their increasing incidence and prevalence. Studies have demonstrated that IRFs are important molecules involved in triggering IFN transcription, which play a critical role in the pathogenesis of NAFLD/NASH (111).

**Figure 3:**
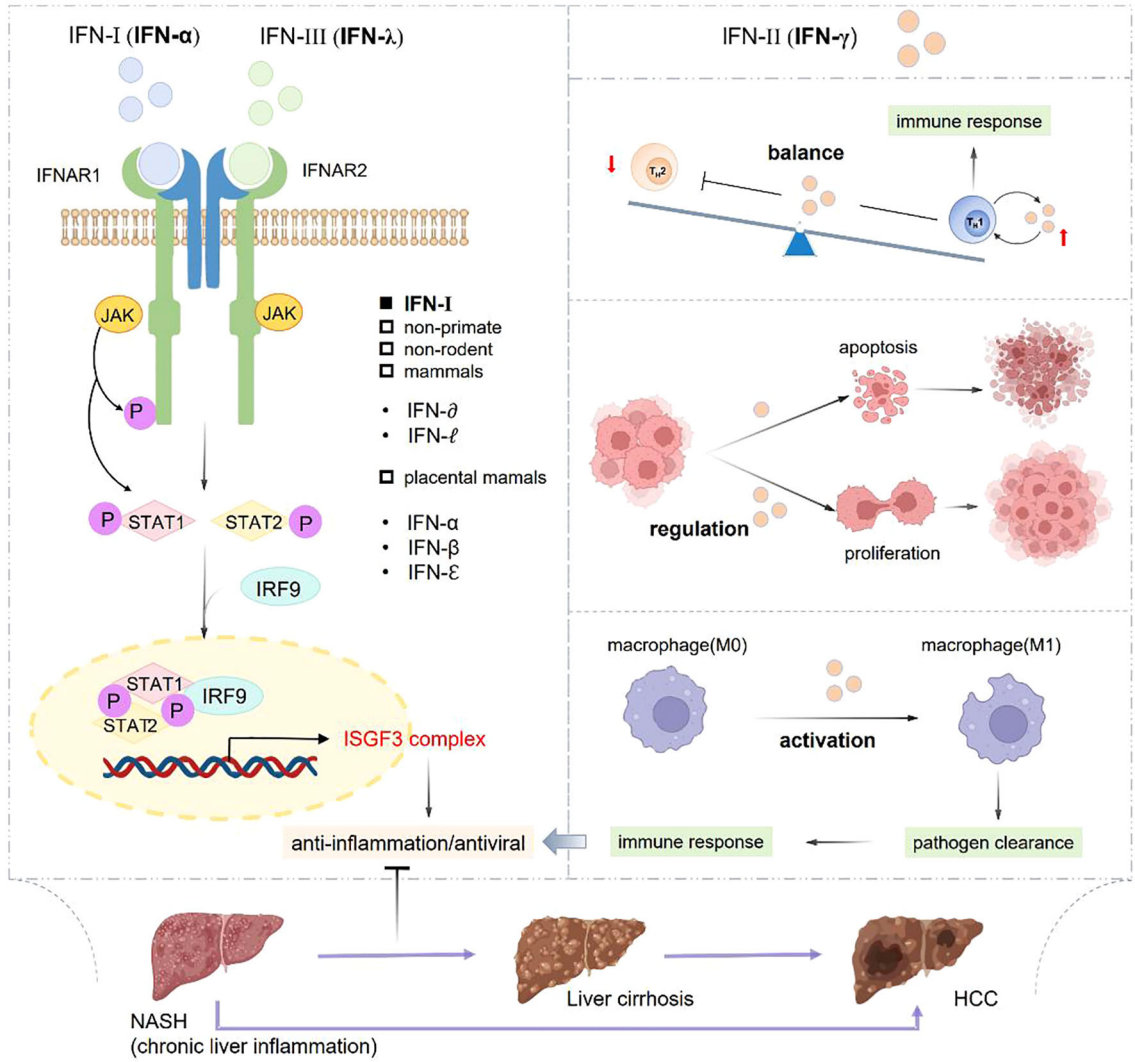
Classification and role of IFNs in inflammation and immune response. IFN-α and IFN-γ respectively activate STAT1 and STAT2 to form into STAT1/STAT2 heterodimer, which can interact with IRF9 to assemble ISGF3 complex, so as to exert antiviral and anti-inflammatory effects. IFN-γ possesses the abilities of promoting macrophage activation, controlling the Th1/Th2 balance, regulating cell apoptosis and proliferation to play important roles in pathogen clearance, anti-virus and anti-inflammation effects.

Type II IFNs, specifically IFN-γ, produced by T helper (Th) cells, which is associated with adaptive immune system. IFN-γ, a pleiotropic cytokine, exhibiting essential effects on mediating Th1 immune responses, enhancing antiviral activity, modulating Th1/Th2 balance and regulating cancer cells apoptosis and proliferation (**Figure 3**). These diverse functions of IFN-γ highlight its significant and potential therapeutic implications in infections, inflammation, and cancers (112).

IFN-α and IFN-λ respectively activate STAT1 and STAT2 to form into STAT1/STAT2 heterodimer, which can interact with IRF9 to assemble ISGF3 complex, so as to exert antiviral and anti-inflammatory effects. IFN-λ possesses the abilities of promoting macrophage activation, controlling the Th1/Th2 balance, regulating cell apoptosis and proliferation to play important roles in pathogen clearance, anti-virus and anti-inflammation effects.

#### 2.2.2 IFN-mediated signaling pathway

Among the IFNs, IFN-γ plays a critical role in mediating immune and inflammation responses through activating the JAK-STAT signaling pathway. IFN-γ/STAT1 pathway is promptly activated responding to infections or inflammatory signals and then being deactivated once the threat is resolved, assisting to maintain the immune balance (113). Moreover, IFN-γ/STAT1 pathway is regulated by facilitating the non-cytolytic virus clearance and modulating the neuronal excitability within neurons, thus contributing to enhance the immune surveillance (114). The stimulator of interferon genes (STING) is emerging as a novel role with pleiotropic effects in the immune system. The applications of STING agonists manifest their importance in cancer immunotherapy. STING signaling is involved in promoting tumor metastasis (115, 116). The STING pathway have prominent effects in detecting the pathogens and self-DNA released from tumor cells, then the antiviral immunity is triggered by activating NF-kB signaling and inducing immune-stimulated genes (ISG) and type I IFN expression (117, 118).

#### 2.2.3 Role of IFN in liver diseases

Currently, main treatments for CHB are IFNs and nucleotide analogues. These reagents are utilized to suppress HBV replication, reduce liver inflammation and prevent disease progression. IFNs exert effects by boosting the immune response against the virus, while nucleotide analogues can interfere with the process of viral replication. Hackstein et al. (119) revealed that IFNs signalings stimulated the myeloid-derived IL-10 expression, presenting a novel avenue for enhancing the T-cell immunity in patients with chronic liver disorders.

Previous studies indicate that Interferon regulatory factor 1 (IRF-1) is crucial in occurrence and evolution of liver diseases, which can inhibit hepatitis virus (A/B/C/E) replication, alleviate liver fibrosis progression, making it a potential mediator for predicting the prognosis and recurrence of liver cancer (120). IRF-1 binds to the promoter of programmed cell death protein ligand 1 (PDL1) in tumor cells and triggers immune escape in HCC. Besides, IRF-1 can promote malignant phenotype of HCC by activating mTOR/STAT3/AKT signal pathway (121). STING is important to maintain the liver homeostasis. When STING pathway is stimulated, IFNs and pro-inflammatory cytokines are produced via downstream IRF3 and NF-kB pathways to inhibit hepatitis virus replication, initiate the immune response, facilitate the oncogenesis and metastasis of HCC (122).

### 2.3 Tumor necrosis factor

TNF-α was first cloned in 1984 followed by the cloning of its receptors TNFR1 and TNFR2 after 1990 (123, 124). Early studies suggested that TNF-α exhibited anti-neoplastic properties (125). With increasing researches on TNF, some experiments have revealed that TNF-α can directly impact on tumor cells and trigger the pro-metastatic property by fostering cancer stem cells (CSCs) generation, promoting epithelial cells transit to mesenchymal cells, enhancing the invasion capability and inducing the metabolic alterations (126).

The inflammatory responses play a key role in innate and adaptive immune systems, which is a protective immune response that maintains tissue homeostasis by eliminating harmful stimuli, including damaged cells, irritants, pathogens and sterile lesions (32). Studies have shown that TNF and its receptors TNFR1 and TNFR2 can be used as therapeutic targets for certain diseases (127). TNF is a pleiotropic cytokine involved in a variety of inflammatory and autoimmune diseases, such as rheumatoid arthritis (RA), psoriasis, Alzheimer’s disease (AD) and multiple sclerosis (MS). TNF-α serves as a soluble toxic medium produced by liver Kuff cells (KC), mediating the inflammation, tumor cell proliferation and apoptosis.

#### 2.3.1 TNF-mediated signaling pathway

TNF was initially identified and named for its anti-tumor properties. Both forms of TNF have biological roles, with sizes of 26 kDa and 17 KDa respectively. TNF can exert its actions in paracrine or autocrine manner by binding to one of two cell surface receptors, TNF receptors 1 and 2 (TNFR1 and TNFR2, named p55 and p75, respectively). Among them, the cytoplasmic region of TNFR1 contains a characteristic death domain (DD) comprised of 80 amino acids, which is utilized to assemble the signaling complex, hence earning it the designation of a death receptor (128). Receptor-interacting protein kinase 1 (RIPK1) binds to TNFR1 to activate NF-kB pathway. TNFR1 signaling can also induce the apoptosis and necrosis through the phosphorylation of receptor interacting protein kinase 3 (RIPK3) and changes of mixed lineage kinase domain-like (MLKL) (129). Guo et al. (130) found that TNF was related to TNFAIP1, TNFAIP3, TNFAIP5, TNFAIP6, TNFAIP8 and TNFAIP9, which could directly participate in TNFAIP1, TNFAIP5, TNFAIP8 and TNFAIP9 activation. The results confirmed that TNFAIP1, TNFAIP2 and TNFAIP3 induced the tumor progression by mediating NF-κB pathway.

#### 2.3.2 Role of TNF in liver diseases

Alcohol metabolism produces toxic metabolites by releasing cytokines, chemokines, and ROS (131). Long-term chronic alcohol abuse can lead to liver injury, hepatitis, fibrosis and HCC. The development of alcoholic liver disease (ALD) can be influenced by TNF and IFN-γ. ALD is a multimodal disease that encompasses alcoholic steatohepatitis (ASH) and alcoholic fatty liver disease (AFLD), ultimately giving rise to liver fibrosis and cirrhosis. IL-6 and IL-10 act as protective cytokines in the liver, which are correlated with ALD progression. NASH is an inflammatory subtype of NAFLD characterized with significantly elevated pro-inflammatory cytokines. TNF-α expression is relevant to the degree of liver damage in ALD and NASH (132). Alcohol consumption may bring about liver inflammation through inducing the translocation of enterogenic endotoxins circulate to portal vein. Moreover, alcohol consumption triggers KCs activation via the lipopolysaccharide/toll-like receptor 4 (LPS/TLR4) pathway, further promoting liver inflammation. HSCs exert a dramatic influence in liver fibrosis angiogenesis. By exploring anti-angiogenic therapy, we’re making efforts to pursue therapeutic approaches to address the alcoholic liver fibrosis (ALF) (133).

Researchers have confirmed that TNF-α is important in the pathogenesis of NAFLD (30). TNF-α is originally discovered to prevent HCC development, it exhibits a gradual increase tendency from normal controls to patients with NAFLD and HCC (134). TNF-α triggers hepatocyte apoptosis, necrosis, liver inflammation and regeneration, autoimmune hepatitis and HCC, making it a promising diagnostic biomarker and appealing therapeutic target for NAFLD-associated HCC (27). Han et al. (135) demonstrated that TNF- inducible gene 6 protein and its derived peptide could ameliorate the liver fibrosis in mice with ALD by restraining CD44 activation, so as to be a treatment approach for ALF. NF-κB signaling plays a dual role in the advancement of liver diseases. It exerts a protective effect and maintains normal liver function under physiological conditions; while under pathological conditions, the excessive activation of NF-κB may promote the hepatocarcinogenesis and tumor progression (136–138). TNF-α can facilitate the occurrence and development of HCC by activating the NF-κB or JNK pathway. It may also hinder the HCC progression through inhibiting hepatocyte apoptosis (139).

Hepatic encephalopathy (HE) refers to a combination of mental and neurological disorders of central nervous system secondary to serious liver diseases. HE is commonly observed in patients suffering from acute or subacute liver injury (severe viral hepatitis, poisoning), cirrhosis and advanced liver cancer, as well as some patients after the operation of transjugular intrahepatic portosystemic stent shunt (TIPS). When liver function is severely impaired, toxic metabolites in the blood cannot be eliminated. The secretion of TNF-α, IL-1β and IL-6 can disrupt neurotransmission, leading to dysfunction of cognitive and motion (140, 141).

### 2.4 Colony stimulating factor

Colony stimulating factor 1 (CSF-1) is involved in recruiting the monocytes from peripheral blood to TME and promoting their differentiation into macrophages. CSF-1 can restain the tumorigenesis of tumor-associated macrophages (TAMs), making it a key regulator of survival and proliferation. The phenotypes of TAMs are categorized into type M1 and M2. M1-type macrophages are generated in response to granulocyte macrophage colony stimulating factors (GM-CSF or CSF2) and stimulated by IFN-γ, lipopolysaccharide, TNF-α, which share the same properties of anti-tumor and pro-inflammation. M2-type macrophages possess biological functions of anti-inflammatory property, regulating immune response, promoting tumor growth, angiogenesis, invasion and metastasis, as well as resisting to cancer treatments.

#### 2.4.1 CSF-mediated signaling pathway

In recent years, CSF1/CSF1R axis has attracted more attention in clinical applications. Fujiwara et al. (142) confirmed that CSF1R inhibitor Pexidartinib (PLX3397) could effectively inhibit the activation of pERK1/2 signaling molecules by CSF-1, resulting in decreased polarization, survival and chemotaxis of TAMs. This pathway could induce the simultaneous depletion of TAMs and Forkhead protein/transcription factor (FOXP3+) Treg cells, hinder the primary tumor growth and distant metastasis, improve the metastasis-free survival, which made a new breakthrough in cancer immunotherapy.

#### 2.4.2 Role of CSF in liver diseases

It’s reported that more than one million people die from advanced chronic liver diseases worldwide every year, and liver surgery remains a crucial treatment option to achieve long-term survival for patients with HCC. Granulocyte colony-stimulating factor (G-CSF) is secreted by fibroblasts, monocytes, macrophages, endothelial cells, stromal cells and bone marrow cells, which can promote the maturation and release of granulocytes, and induce the synthesis of macrophages and eosinophils. Previous researches have shown that G-CSF can regulate the inflammatory response, enhance the liver regenerative capacity, and improve the survival rates of patients with advanced chronic liver disorders. In 2023, Colli et al. (143) conducted 20 experiments involving 1,419 participants from different countries. The intervention measures of this research included administering G-CSF alone or combination with any of following factors: growth hormone, erythropoietin, N-acetylcysteine, infusion of CD133-positive hematopoietic stem cells or autologous bone marrow monocytes. The results demonstrated that utilizing G-CSF alone or combination with above factors was beneficial for participants who experienced one or more liver disease-related complications, such as HE, hepatorenal syndrome and esophageal varices rupture bleeding. Moreover, G-CSF reduced the mortality and development of infections like sepsis in patients with decompensated chronic liver diseases.

In 2019, Zhu et al. (144) isolated chemical-induced liver tumors from wild-type mice and OPN- knockout mice, tumor infiltrating cells and inflammatory immunoprofiles in the two groups were analyzed respectively, and then they conducted a cell co-culture experiment *in vitro*. The results indicated that OPN/CSF1/CSF1R axis exhibited immunosuppress property in HCC. Furthermore, TAMs migration could be impeded by blocking the CSF1/CSF1R axis, thereby enhancing the efficacy of immune checkpoint inhibitors in treating HCC. This provides a new method for HCC clinical treatment.

### 2.5 Chemokines

CKs, secreted by leukocytes and stromal cells, possessing the characteristics of chemotaxis and activation. Based on the arrangement position of first two cysteine residues near the amino terminal, CKs are divided into four subfamilies, including CXC, CC, CXXXC and C subfamily (145). After binding to specific receptors, CKs activate the phosphatidyl creatine kinase or phosphatase pathway to trigger leukocyte adhesion and regulate liver inflammation by controlling the migration of hepatocytes, KCs, HSCs and immune cells.

#### 2.5.1 CK-mediated signaling pathway

CKs are crucial in promoting tumor cell invasion with tumor infiltrating lymphocytes (TILs), and recruiting TAMs (146). There are 14 typical chemokine receptors that recognize multiple ligands, which can bind to multiple receptors in a similar manner (147). CKs and their receptors regulate tumor cells proliferation and metastasis through various signaling pathways (148). Leukocyte-derived chemotaxin-2 (LECT2), secreted by hepatocytes, which is involved in pathogen clearance, inflammation and immune response, tumor metastasis in NASH (149).

IL-8 has two receptors, IL-8RA and IL-8RB, also known as CXCR1 and CXCR2, the sequence homology of which is up to 77% (150). CXCL8 plays an important role in inflammatory diseases and tumors (151). Zhai et al. (152) found that CXCL8 might cause the chemotherapy resistance of gastric cancer through activating the NF-κB signaling and up-regulating the ATP-binding cassette subfamily B member 1 (ABCB1). CXCR1 only binds to CXCL6 and CXCL8, while CXCR2 is a typical G protein-coupled receptor responding to CXCL1, CXCL2, CXCL3, CXCL5, CXCL6, CXCL7 and CXCL8 (153). CK receptors and ligands have a profound effect on the occurrence, angiogenesis, metastasis, proliferation and invasion of lung cancer, colorectal cancer, gastric cancer and HCC, making them promising targets for immunotherapy, which impact the prognosis and treatment outcomes of patients with tumors (154, 155).

By modulating the pathways mediated by CKs and their receptors, TME immunophenotype can be reshaped to improve the immunotherapy efficacy. Desurmont et al. (156) discovered that CXCR2 and CXCL7 overexpression shortened the overall survival (OS) and disease-free survival (DFS) of colorectal cancer patients with liver metastasis, which could be recognized as a predictor of poor prognosis in metastatic colorectal cancer. Hermitte et al. (157) demonstrated that LECT2 low expression in HCC was relevant with advanced tumor grade and immune invasion, which makes it a promising biomarker in HCC immunotherapy.

CCL28, a ligand of CCR3/CCR10, makes some effects on the growth, metastasis and spread of breast cancer. CCR10/CCL27 signaling is associated with the adherence and survival of melanoma cells during the metastatic process (158). CXCR5/CXCL13 is related to bone metastasis of prostate cancer (159). Zanetti et al. (160) proved that recombinant CXCL13 could increase cell proliferation of pAKT and B-cell acute lymphoblastic leukemia (B-ALL), CXCR5/CXCL13 axis might be considered as a prognostic marker and promising target for treating the prostate cancer and B-ALL.

#### 2.5.2 Role of CK in liver diseases

ALD is mainly correlated with excessive alcohol consumption. If ALD isn’t well controlled, it may evolve into liver fibrosis, cirrhosis, eventually HCC with fatal property and poor prognosis. There is substantial evidence showing that therapeutic agents targeted to oxidative stress or gut-liver axis are considered as crucial treatments for ALD by suppressing inflammatory response and enhancing liver regeneration (161–163). Alcohol consumption promotes hepatocytes to secrete CKs, in turn triggering the inflammatory cells to recruit to liver and increasing micro ribonucleic acid (miRNA)-155 expression through NF-κB pathway, then stimulating the lipopolysaccharids-triggered KCs to secrete TNF, ultimately facilitating liver inflammation (164, 165). Therefore, it’s essential to search for therapeutic strategies to block the CK-mediated pathway for treating ALD and HCC.

NAFLD is the third leading cause of HCC worldwide and characterized by steatosis, liver inflammation, liver cell damage and progressive fibrosis, which can be divided into nonalcoholic fatty liver disease (NAFL) and NASH (166). Several cellular components or molecular pathways of NASH can be targeted simultaneously to achieve anticipative therapeutic goals (167–171). CCL2/CCR2 is utilized as therapeutic targets for treating NASH (172). The gut-liver axis is critical in NAFLD pathogenesis. The intestinal flora of patients with NAFLD, cirrhosis and HCC are significantly associated with systemic inflammation. The contents of IL-8/13, CCL3/4/5 are higher in HCC than those in normal subjects (173). Immune cells mainly include macrophages, monocytes and neutrophils, derived from hematopoietic stem cells of bone marrow, which are responsible for immune defense, stabilization and immune surveillance (174). Macrophages and hepatocytes can release CKs, and immune cells are attracted into the injured position to promote collaborative recruitment (175). CXCL16 can promote cell proliferation, invasion and migration by mediating PI3K/AKT/PKB and ERK/MAPK pathway, which plays a key role in the progression of NAFLD, cancer, atherosclerosis, renal fibrosis (176).

Excessive alcohol consumption, a high-fat diet, and chronic hepatitis virus infection can lead to liver inflammation, eventually cause portal hypertension, liver fibrosis, cirrhosis, HCC. Endothelial p300 is a regulator of gene transcription with the properties of activating NF-κB pathway, promoting CCR2+ monocytes/macrophages accumulation and increasing CCL2 expression in the damaged liver, ultimately resulting in portal hypertension and liver fibrosis (177). Guo et al. (178) discovered that cenicriviroc could inhibit liver fibrosis and cirrhosis by inactivating CCR2-STAT1/NF-κB/ERK pathway. Puengel et al. (179) demonstrated that CCR2/5 signaling ameliorated liver fibrosis through inhibiting the monocytes and macrophages. Dudek et al. (180) showed that CXCR6^+^ CD8 T cells were abundant in NASH, characterized by low-activity transcription factor forkhead box O1(FOXO1). Fas and its ligand FasL were related to cell apoptosis. Blocking the FasL could prevent self-attack of CD8 *in vitro* and T cells after adoptive transfer *in vivo*, which further improved the liver injury in NASH.

HCC, one of the common solid malignancies, the key factors of which are alcohol intake, sex, age, lipid, obesity, type 2 diabetes, dysregulation of gut microbial and genetic variation. Previous experiments and theories have confirmed that CXCR1/2 axis activated immunosuppressive cells, and CXCR3/4 axis increased the recruitment of effector cells. TME immunophenotype might be reshaped to improve the efficacy of tumor immunotherapy by regulating CKs and CK receptor-related pathways (181). As is known to all, forkhead frame transcription factor C1 (FOXC1) is closely related to blood vessels maturation through interacting with Notch and VEGF pathway. Hang et al. (182) found that CXCR1 and CCL2 could target to FOXC1, making them predictors of postoperative recurrence and OS in HCC.

CCL20, expressed in liver, colon, skin, the specific receptor of which is CCR6. CCL20/CCR6 axis can facilitate cell proliferation and migration by modulating immune cells to reshape TME, so as to be utilized as a target for immunotherapy in HCC, colorectal cancer, breast cancer, pancreatic cancer, cervical cancer and kidney cancer (183). In 2023, Xie et al. (184) found that the overexpression of E-26 specific sequence variant 4 (ETV4) in hepatocytes transactivated the expression of PD-L1 and CCL2, consequently inhibited CD8+T cell accumulation. Knockout the CCL2 gene with lentivirus or CCR2 inhibitor CCX872 could compromise the infiltration of TAM and MDSC induced by ETV4 and HCC metastasis, which provided a theoretical basis for developing a novel combined immunotherapy strategy for HCC. We summarize the mechanisms and clinical applications of chemokine receptors or ligands in **Table 3**.

**Table 3:** The mechanisms and clinical applications of chemokine receptors or ligands in liver disease.

Chemokine receptor or ligand	Mechanism	Disease type	Author
CXCR1/CCL2	Promoting cells proliferation, migration and invasion	HCC	Hang et al. (182) Xie et al. (184)
CCR2/5	Inhibiting liver fibrosis and cirrhosis by mediating CCR2 -STAT1/NF-κB/ERK pathway; Restraining the circulating Ly6C+ monocytes and macrophages derived from liver monocytes	Liver fibrosis and cirrhosis; NASH	Guo et al. (178) Puengel et al. (179)
CCR2/CCL2	Promoting the infiltration and metastasis of TAM and MDSC; Recruiting the monocytes/macrophages and activating KCs; Promoting tumor cell angiogenesis and metastasis	NASH; HCC	Zhuang et al. (185)
CXCR6	Contacting CXCR6 CD8+ T cells with ATP led to the up-regulation of FasL; Blocking the FasL could sustain the self-attack of CD8 *in vitro* and T cells after adoptive transfer *in vivo*, and further ameliorated the liver damage	NASH	Dudek et al. (180)
CXCL16/CXCR6	Leading to tumor cells proliferation, migration, invasion and metastasis by mediating the PI3K/AKT/PKB or ERK/MAPK pathway	NAFLD	Korbecki et al. (176)
CCL20/CCR6	Regulating immune cells to reshape TME; Promoting cell proliferation and migration	HCC	Kadomoto et al. (183)
Leukocyte cell-derived chemotaxin-2 LECT2	Involved in pathogen clearance, inflammatory and immune responses, tumor metastasis; In HCC with low expression of LECT2, β-catenin pathway was activated to induce epithelial cell transform into mesenchymal cell, then triggered TME and tumor phenotype remodel, ultimately inhibited HCC occurrence and progression	NASH; HCC	Takata et al. (149) Hermitte et al. (157)

### 2.6 Growth factor

#### 2.6.1 GF-mediated signaling pathway

As a potential signaling molecule, GF plays a dramatic role in the repair of tissue and the regeneration of vascular through regulating the cell growth, stem cell differentiation and tissue healing. Llopis-Hernández et al. (186) proved that fibronectin(FN) was utilized to make the GF interacting with the extracellular matrix (ECM) proteins, which could offer a new substrate for efficient and low dose delivery to local lesions. The polymer system might reduce the dose of GF and locally delivered GF to the site where regeneration was needed. By targeting the integrins and GF receptors, the synergistic effect might be maximized. The integrin/GF signaling contributed to the differentiation of stem cell and the repair of tissue.

GF is a crucial component of regenerative strategies for therapeutic repair or tissue replacement. There are some complex signaling pathways in mediating the liver regeneration (**Table 4**). Chen et al. (198) discovered that controlling the tissue development by modulating the local availability of GF combinations would provide a powerful tool for studying and manipulating a wide range of developmental and regenerative process. The optimal GF combination was delivered to the target site safely. GFs can be classified into PDGFs, bone morphogenetic proteins (BMPs), insulin-like growth factors (IGFs), TGF-β and VEGF, which have good prospects in application for the bone healing and osteogenesis (199).

**Table 4:** Signaling pathway mechanisms in liver regeneration phase.

Signaling pathway	Main mechanism	Author
NO pathway (initiation phase)	Activating soluble guanylyl cyclase (sGC)/cyclic guanosine monophosphate (cGMP)/cGMP-dependent protein kinase 1 (PKG-1) pathway, releasing NO by endothelial cells, then initiating liver regeneration	Emily et al. (187) Dai et al. (188)
YAP pathway (initiation phase)	Increasing YAP nuclear level and gene expression at initial stage, resuming at the termination stage, thereby regulating the hepatocyte proliferation	Grijalva et al. (189)
IL-6 pathway (initiation phase)	Stimulating IL-6/JAK/STAT3 pathway; Regulating IL-6, STAT3, c-Myc and c-Jun signaling molecules	Wen et al. (190)
TNF-α pathway (initiation phase)	Activating NF-κB pathway	Zhang et al. (191)
Notch pathway (initiation phase)	Activating Notch- HIF-1a pathway, promoting LR by enhancing hepatocytes proliferation	Zhang et al. (192)
Wnt/β-catenin pathway (initiation phase)	Inducing the transformation of transitional liver progenitor cells (TLPCs) into hepatocytes	Pu et al. (193)
HGF pathway (initiation phase)	HGF/c-Met signaling can regulate the survival of liver progenitor cells; Activating downstream pathways such as PI3K/AKT, JAK/STAT3 and Ras/Raf pathways	Chiang et al. (194) Li et al. (195)
TGF-β pathway (termination phase)	Activating TGF-β- Smad 2/3 signaling pathway, thereby inhibiting cell cycle	Li et al. (196)
PI3K/AKT/mTOR pathway (progression phase)	Panax notoginseng saponins (PNSs) promoting LR through activating PI3K/AKT/mTOR pathway	Zhang et al. (197)

Cytokines and GFs can regulate cell recruitment, migration, adhesion, proliferation, differentiation and apoptosis. Previous researches have demonstrated that gut microbiota can modulate the release of IL-6, TNF-α, hepatocyte growth factor (HGF), IFN-γ and TGF-β, which are involved in liver regeneration and different liver disorders (200, 201). Tumor cells have the ability to produce the cytokines to facilitate cell growth and overexpress the GF receptors. Abnormal GF signalings can improve the survival through mediating RAS/RAF/MEK/ERK and PI3/AKT/mTOR pathways, and some GFs may boost tumor cell growth and metastasis by post-transcriptional mechanisms (202).

#### 2.6.2 Role of GF in liver diseases

The liver has a satisfactory capacity of regeneration, so that it can recover the function and size even after 70% of the liver is partially removed. The process of liver regeneration involves a complex network of hepatocyte growth-promoting factors, cytokines, signaling pathways and transcription factors. The mechanical and chemical environment of the liver changes with the accumulation of various GFs and the remodeling of the extracellular environment. Liver regeneration occurs when the liver is damaged by viruses or drugs, as well as after partial hepatectomy or liver transplantation. Some characteristic alterations will happen after partial hepatectomy or partial liver transplantation, such as hemodynamic changes in portal vein flow pressure, tissue ischemia and hypoxia, and platelet activation (203). The process of liver regeneration is a cascade chemical signaling pathway. Signaling molecules are delivered to the nucleus to activate the liver regeneration, including hepatocyte proliferation, stem cell differentiation, extracellular matrix remodeling, and termination signals that regulate the size of the regenerated liver. KCs and HSCs can secrete transforming growth factor β (TGF-β1) and HGF. The KCs can activate the Wnt signaling pathway to act on the hepatocytes. The signal transduction mechanisms involved in the liver regeneration mainly including NO pathway, YAP pathway and actomyosin remodeling (204). Moreover, following signaling pathways like IL-6, TNF-α, Notch pathway, Wnt/β-catenin, HGF, TGF-β pathway and PI3K/AKT/mTOR pathway are also involved (191). During the process of liver regeneration, HGF triggers the loss contact of cell-matrix, thus promotes the hepatocytes proliferation.

Myelogenic growth factor is a mechanically-induced vascular secretion signal present in the human liver endothelial cells. By activating MAPK and STAT3 signaling, primary human hepatocytes from different donors are induced to improve their survival (205). The liver acts as a central immune organ that activates the immune system in response to the circulating antigens (206, 207). Di-Iacovo et al. (208) revealed that optimal liver regeneration was achieved by integrating the IL-6/JAK/STAT3 and PI3K/PDK1/AKT pathways to accelerate the cell proliferation.

## 3 Conclusions

**Figure 4:**
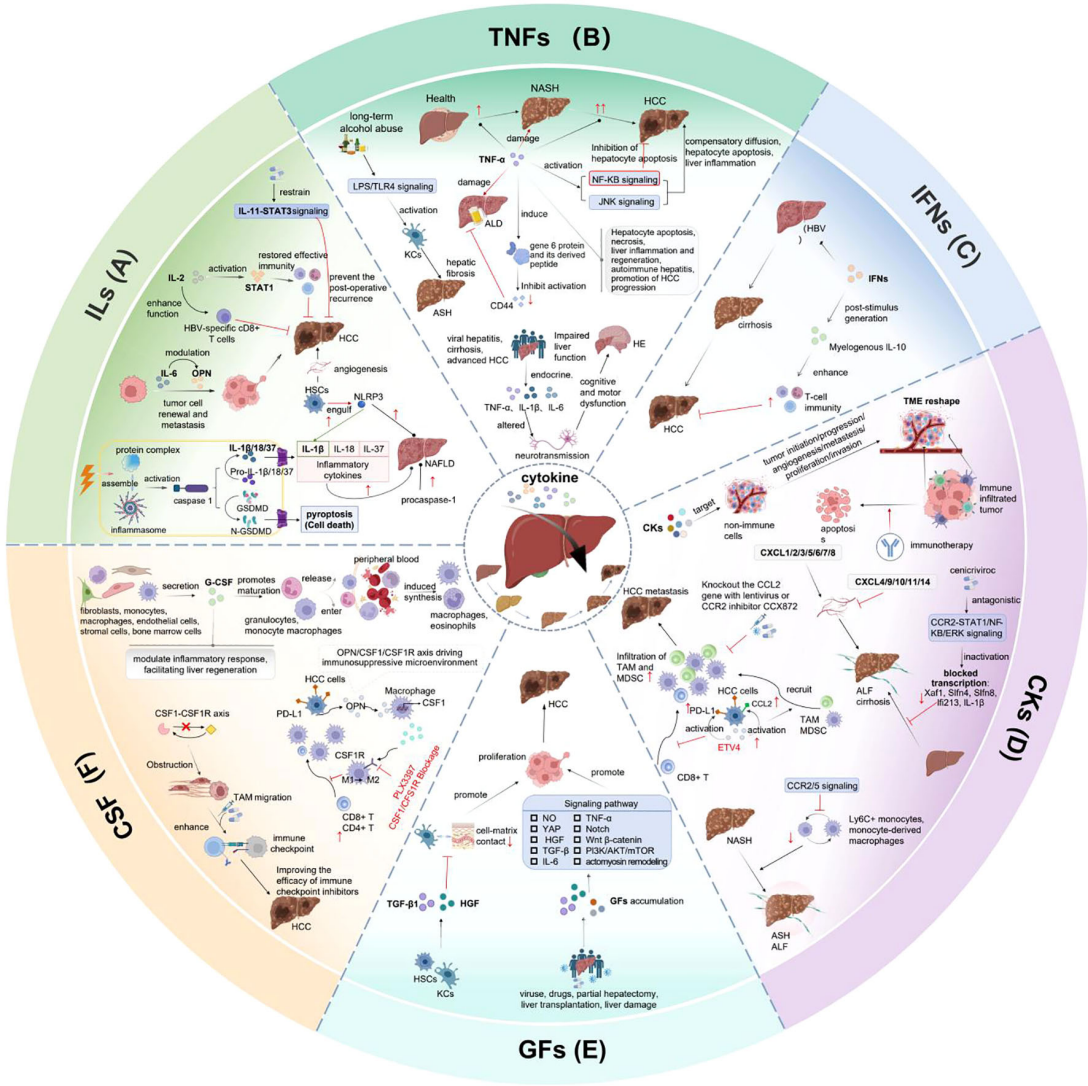
Pivotal role of cytokines in liver disease pathogenesis and clinical application. **(A)** IL-2 can activate STAT1 signaling, restore effective immune and enhance HBV-specific CD8+ T cells. IL-6 impacts tumor cell renewal and metastasis by modulating OPN. The postoperative recurrence of HCC can be prevented by constraining IL-11-STAT3 signaling. HSCs engulf NLRP3, enhance IL-1β expression, cause liver injury, fibrosis and HCC. **(B)** TNF-α affects liver inflammation, apoptosis by activating NF-κB and JNK pathways. HE is appeared in patients with acute or subacute liver injury, cirrhosis and advanced HCC. **(C)** IFNs can suppress HBV replication, reduce liver inflammation, prevent disease progression by stimulating myeloid-derived IL-10 expression and enhancing immune response. **(D)** CKs promote tumor initiation, progression, angiogenesis, metastasis, proliferation and invasion by targeting the non-immune cells and reshaping the TME. ETV4 overexpression in hepatocytes can activate PD-L1 and CCL2, increase TAM and MDSC infiltration, inhibit CD8+T cell accumulation, then promote HCC metastasis. **(E)** When liver function is damaged by viruses, drugs, as well as after partial hepatectomy or liver transplantation, Liver regeneration occurs with GFs accumulation and extracellular environment remodeling through activating pathways including NO, YAP, actomyosin remodeling, IL-6, TNF-α, Notch, Wnt/β-catenin, HGF, TGF-β and PI3K/AKT/mTOR pathways. **(F)** G-CSF is secreted by fibroblasts, monocytes, macrophages, endothelial cells, stromal cells and bone marrow cells. It can induce macrophages and eosinophils synthesis, regulate inflammatory response and enhance liver regeneration. OPN/CSF1/CSF1R axis has immunosuppress property in HCC microenvironment.

Cytokines are essential for biological processes including cell proliferation, tissue repair, aging, inflammation and immunity. Current researches have demonstrated that TNF, IL-1α/β, IL-1Ra, IL-6, IL-18, IL-33, IL-36, IL38, CCL2 and CCR2 are closely associated with liver disorders (209–211). The pivotal roles of cytokines in liver diseases pathogenesis and clinical application are elaborated respectively in the previous sections of this review (**Figure 4**).

Nowadays, recombinant IL-2, IFN-α and TNF are applied into cancer immunotherapy (212, 213). The therapeutic efficacy is dramatically hindered by complex pleiotropy, redundancy, toxicity, off-target effect, short half-life and narrow therapeutic window of cytokines (214). Moreover, the therapy-induced immune response may limit the efficacy by neutralizing antibodies with drugs or restrict the security through inducing inflammatory responses (215). Based on these factors above, there are still great challenges in developing innovative drugs based on cytokine therapy.

Supercytokines are formed into fusion proteins or bifunctional cytokines by modifying the binding domains, enhancing the affinity or improving the half-life of cytokines. There are also adaptor cytokines, synplastic cytokines, nanocellular cytokines, adaptive immune cells equipped with cytokines, and cytokine-armed oncolytic viruses existing in the clinical applications (216). Deckers et al. (217) developed safe and effective cytokine-based therapies for immune-mediated diseases by means of technological innovations in protein engineering, nanomedicine, RNA technology and cell engineering. In addition, prodrugs of various cytokines including IL-2, IL-12 and IFNα2b were exploited to treat cancers (218, 219).

Recently, immunotherapy has emerged as promising treatment in solid cancers, such as immune checkpoint inhibitors (ICIs), tumor vaccines, oncolytic virus immunotherapy, adoptive cell therapy and cytokine therapy (220–222). The combination of ICI and VEGF inhibitor is verified to be a first-line therapy for advanced HCC (223). Meanwhile, antiviral therapy is necessary during the process of HCC treatment (94). The recurrence and metastasis rate can be reduced when the surgery is combined with IFN therapy mediated by cGAS-STING pathway (224). Moreover, cGAS-STING signaling pathway agonists can also be combined with radiotherapy, chimeric antigen receptor T Cell (CAR-T) therapy, oncolytic virus therapy to enhance tumor immunity and improve efficacy (225, 226). By means of exploring the mechanisms of cytokines and constructing the diverse combination complexes to inhibit the process of liver diseases, we are trying to pursue more promising targets for immunotherapy strategies in liver disorders.
